# Association of oral health status and salivary profile of rheumatoid arthritis disease subjects and healthy subjects and prediction of caries risk using cariogram- A cross-sectional analytical study

**DOI:** 10.1016/j.jobcr.2025.03.020

**Published:** 2025-03-31

**Authors:** Ananya Jha, Aparna K S, Praveen Jodalli, Avinash B R

**Affiliations:** a Manipal College of Dental Sciences Mangalore, Manipal Academy of Higher Education, Manipal, Karnataka, 576104, India; b Centre for Dental Education and Research, All India Institute of Medical Sciences, New Delhi, India; c Department of Public Health Dentistry, Manipal College of Dental Sciences Mangalore, Manipal Academy of Higher Education, Manipal, Karnataka, 576104, India

**Keywords:** Caries risk, Cariogram, Oral health, Rheumatoid arthritis, Saliva, Health and wellbeing

## Abstract

**Background:**

Rheumatoid arthritis (RA) is a persistent inflammatory condition that predominantly affects the joints. However, it also affects the oral health, including temporomandibular joint abnormalities, periodontal disease, and xerostomia. Although these correlations exist, there is little proof that RA is associated with certain markers of dental health. The purpose of this study is to close this gap by assessing the salivary profile and oral health status of RA patients.

**Aim:**

To assess and contrast the salivary profile and oral health status of people with rheumatoid arthritis with that of healthy controls.

**Objectives:**

To evaluate.

1. Periodontal health and caries experience using WHO guidelines Adult oral health proforma (2013)

2. Salivary parameters using a salivary kit.

3. Cariogram-based assessment of caries risk.

4. Association between Rheumatoid arthritis and Oral health.

**Methods:**

A total of ninety volunteers were enrolled, forty-seven of whom were age-matched healthy individuals and forty-three of whom were patients with rheumatoid arthritis. A structured questionnaire, clinical assessment, and saliva collection were used in the data gathering process. Dental health was evaluated using the WHO Oral Health Assessment Form for Adults (2013), and salivary flow rate and pH were monitored. The Reduced Cariogram model was used to assess the risk of caries. SPSS version 22 was used for the statistical analysis, with significance set at p < 0.05.

**Results:**

Compared to controls, those with RA had significantly higher rates of dental caries, more gingival bleeding, and deeper periodontal pockets (p < 0.05). Patients with RA also showed decreased salivary pH and decreased stimulated and unstimulated salivary flow rates. Salivary flow rates were found to be negatively correlated with pocket depth, gingival bleeding, and dental caries. Furthermore, the Cariogram showed that patients with arthritis had a higher incidence of dental caries.

**Conclusion:**

The findings suggest that dental health is negatively impacted by rheumatoid arthritis. Patients with RA have a higher prevalence of dental caries and periodontitis likely due to impaired salivary profile. These results highlight the significance of thorough oral health evaluations and customized preventive methods for people with rheumatoid arthritis in order to lessen oral health issues.

## 1 Introduction

Rheumatoid arthritis (RA) is a systemic polyarticular, degenerative, and inflammatory chronic disorder. It is an autoimmune response that results in inflammation of the synovial membrane and cause potential disruptions to the function of the afflicted joints.^1^ About 0.5 %–1 % of people have RA, and women are three times more likely than men to have the condition.^2^^,^^3^ Several oral manifestations of rheumatoid arthritis have been linked to the disease, including xerostomia, secondary Sjögren's syndrome, temporomandibular joint disorders, and periodontal disease (PD).^2^

Along with its well-established effects on joint health, RA has also been linked to a growing number of oral health issues, such as xerostomia, secondary Sjögren's syndrome, temporomandibular joint disorders (TMD), and periodontal disease (PD).^3^^,^^4^ Among these, periodontal disease has attracted a lot of interest because of its possible reciprocal association with RA. As evidenced by studies by Russell et al.^5^ and Chappell,^6^ RA patients may have a higher prevalence and severity of periodontal disease when compared to healthy people. Patients with RA frequently have higher attachment loss, probing depth, and alveolar bone loss, which contributes to greater breakdown of periodontal tissue and periodontal inflammation.^7^^,^^8^ Porphyromonas gingivalis and other periodontal infections may contribute to the pathophysiology of RA by inducing citrullination and autoantibody production, but there is also evidence that PD may worsen RA by causing systemic inflammation.^9^^,^^10^

Salivary dysfunction is another important but little-studied component of oral health in RA patients, in addition to periodontal disease. Nearly 50 % of people with RA experience xerostomia, or the subjective feeling of dry mouth, which can be caused by secondary Sjögren's disease or medication-induced hyposalivation.^11^ By making people more vulnerable to dental cavities, mucosal infections, and poor wound healing, decreased salivary flow and changed salivary composition can jeopardize oral health.^12^^,^^13^ According to Russell and Reisine,^5^ xerostomia in RA patients may be linked to both medication-induced and disease-related variables, which could further deteriorate oral health. Moreover, systemic diseases such diabetes, obesity, and smoking—all of which are major comorbidities in RA—may make these individuals even more susceptible to tooth caries and periodontal disease.^12^

Dental caries is thought to be a complex microbial illness. Recent research has shown a strong correlation between RA and a higher incidence of dental cavities.^7^, ^8^, ^9^, ^10^ According to Mehdipour et al.,^8^ RA patients have a worse quality of life in relation to oral health, with a higher prevalence of dental caries and a more severe form of the disease. In a similar vein, Sánchez-Medrano et al.^9^ discovered that dental caries was more common in newly diagnosed RA patients than in healthy controls. These results were further supported by Martinez-Martinez et al.,^10^ who showed that RA patients have greater levels of cariogenic bacteria in addition to a higher prevalence of dental caries.^10^ The necessity of focused oral health interventions in RA populations is highlighted by these findings.

The risk assessment is a crucial step in making choices about dental caries prevention and management. A cariogram can be a useful informative means to aid in patient education and encouragement as well as a useful method for dentists to assess caries risk in clinical practice.^11^^,^^12^ The complete and reduced versions of Cariogram are the two versions that are accessible. To provide a thorough caries risk profile, the full Cariogram uses nine caries-related parameters. It can be used as a risk model and predictor to help with intervention planning. With the reduced Cariogram, however, a streamlined caries risk profile is established by using seven caries-related parameters.^14^ The scope of the Cariogram in determining caries risk, especially in young individuals, was highlighted by a few studies.^12^^,^^14^^,^^15^

Studies assessing caries risk in RA patients using standardized assessment tools are scarce, despite growing evidence of a relationship between RA, salivary function, and oral health. To our best knowledge, this is the first study to predict caries risk in RA patients using cariogram. The objective of this study is to assess the caries risk using the Reduced Cariogram model and compare the salivary profile and oral health status between patients with rheumatoid arthritis and healthy controls.

## 2 Methodology

### 2.1 Study setting

A cross sectional analytical study was carried out at a tertiary care hospital's Department of Medicine, Wenlock Hospital and Department of Public Health Dentistry and Department of Oral Medicine and Radiology, Karnataka, India. Ninety participants, aged 18 and above, were enrolled in the study: forty-three RA patients and forty-seven healthy controls. Data collection took place between March 1, 2023, and March 1, 2024, following institutional and ethical approval.

### 2.2 Participant recruitment

RA patients were recruited from the outpatient and inpatient departments of the hospital. while controls were selected from bystanders who met eligibility criteria.

#### 2.2.1 Sample size

Based on the mean differences in oral health indices published by Martinez et al.,^16^ the sample size was determined using G∗Power analysis, taking into account a power of 80 % and a significance threshold of 5 %. Each group had to include at least 42 participants, and each group had 45 people recruited to accommodate for possible attrition. However, the final sample size consisted of 43 RA patients and 47 healthy controls because of unforeseen constraints including participant dropout, exclusion based on eligibility requirements, or recruitment difficulties. The sample is nonetheless statistically sound and comparable enough for useful analysis in spite of this small difference.

### 2.3 Sampling procedure

A consecutive sampling method was used to choose the participants. Participants were recruited from among the patients and bystanders based on the eligibility criteria.

### 2.4 Eligibility criteria for rheumatoid arthritis patients

Eligibility criteria for RA patients included walk-in and in-patients aged 18 years and above who have been diagnosed with Rheumatoid Arthritis and symptomatic for ≥3 months. The diagnosis of RA was confirmed by a rheumatologist based on clinical presentation and laboratory parameters. The diagnosis was based on medical history, physical examination (clinical features such as swelling, redness, and warmth), and blood tests (elevated erythrocyte sedimentation rate (ESR) or C-reactive protein (CRP) level, rheumatoid factor, and anti-cyclic citrullinated peptide (anti-CCP) antibodies) that indicated the presence of an inflammatory process in the body. Participants who were willing to participate and had no other systemic diseases were included. Additionally, they participants have at least 20 natural teeth in their permanent dentition were included. Individuals with adverse habits like smoking and alcohol consumption, those undergoing orthodontic treatment, or using intraoral artificial prostheses were excluded from the study.

### 2.5 Criteria for rheumatoid arthritis

The 1987 American Association of Rheumatology criteria, which require presence of symptoms for at least 3 months and that the patients meet at least four of the seven criteria listed below, are used to diagnose RA: stiffness in the morning in and around joints that persists for at least an hour before becoming fully better; soft tissue swelling (arthritis) of three or more joint areas noted by a doctor; the presence of rheumatoid factor, symmetric swelling (arthritis), rheumatoid nodules, radiographic erosions, and/or periarticular osteopenia in the wrist, as well as hand, or metacarpophalangeal joints.^17^

### 2.6 Eligibility criteria for healthy participants

Based on a thorough case history, participants who were matched for age and gender and free of systemic illness were included. Individuals with any negative habits, such as alcoholism or smoking, were not allowed to participate.

### 2.7 Ethical considerations

The Institutional Ethical Committee provided ethical clearance (IEC Protocol Ref Number: 23005). The respective departments were consulted in order to obtain the necessary permissions. All participants gave their informed consent after being fully informed about the procedure's goal. The World Medical Association Declaration of Helsinki was followed in the conduct of the study.

### 2.8 Examiner training and calibration

To achieve a satisfactory degree of reproducibility, the trainee examiner (AJ) was trained and calibrated by a benchmark examiner (AK), also known as the "Gold Standard." In order to accomplish an acceptable level of agreement (Kappa = 0.85), the trainee examiner covered clinical diagnosis, criteria, recording, and other errors with the ten rheumatoid arthritis subjects who were examined. To ensure inter-examiner reliability, every patient was checked by both the investigator and the examiner.

### 2.9 Study tool and data collection

Expert consensus and validated tools were used to build the structured proforma. It contained sections on demographic information, medical history (including medication usage and duration of illness), oral examination using the WHO Oral Health Assessment Form for Adults (2013), and the Reduced Cariogram model for assessing caries risk. Expert review was used to guarantee content validity, and a pilot study was used to verify reliability. A good internal consistency (α) of 0.85 was obtained. A single, calibrated investigator conducted a clinical examination after administering the structured questionnaire to gather data, and a trained assistant then recorded the results. To avoid confounding from diurnal variation, every patient had their saliva sampled during 9 and 11 a.m. on the same day. Patients were told to sit in a comfortable position upright for 5 min after receiving a graded obtaining cylindrical tube. They were explained the drooling method, which involves drooling saliva into a tube without swallowing for 5 min and the same was used for sample collection.

Salivary profile estimation was applied to collected saliva, which includes salivary flow rate and pH. The study subjects were given a rubber band of standard measurements, and instructed to chew on it. Saliva secreted within the very first minute was to be swallowed, and during the next 5 min, it was to be spit into a test tube with a graduated opening. In millilitres per minute, the flow rate was expressed. Using unstimulated saliva, pH strips were used to measure the salivary pH. The study participants were instructed to expectorate any collected saliva into the specimen gathering cup. The pH strip was immersed in the saliva for 10 s. The color of the strip was compared to a color indicator chart, where healthy saliva is indicated by a green tint. The colors red and yellow, respectively, indicated the acidity of the saliva.

The WHO Oral Health Assessment Form for Adults, 2013 was used to assess the oral health (DMFT index, gingival bleeding and periodontal index) of the study participants. Periodontitis was defined according to the community periodontal index (periodontal probing depth ≥4 mm). Reduced Cariogram was used for caries risk prediction.^12^^,^^15^ After eliminating bacteria, caries-related factors such as susceptibility, diet, saliva, and overall health were scored using an established protocol.^14^

### 2.10 Statistical analysis

Version 22 of the statistical software for social science (SPSS) was used to evaluate the data. When the p value was less than 0.05 (with a 95 % confidence interval), it was deemed significant. Descriptive analysis, comparing mean (SD) and percentage (proportion) was done for continuous and ordinal data respectively. The data was tested for normality and found to be normally distributed. Chi Square/Fisher exact test was used to find out differences between proportions. Mann-Whitney test, a non-parametric test was used to find difference in mean scores as the data was found to be not normally distributed when tested for normality. A multivariate analysis was conducted to measure the associations between RA and oral health status after adjusting for potential confounders.

## 3 Results

In this study, 47 healthy participants (controls) and 43 patients with rheumatoid arthritis (RA) were involved. We recruited subjects based on the criteria and carried out consecutive sampling. The RA group's mean age was 55.0 ± 10.22 years, while the control group's mean age was 38.57 ± 11.51 years. The ratio of female to male participants was higher. There was no statistically significant difference in age (p = 0.21) or gender (p = 0.21) between the research groups (Table 1).

**Table 1:** Distribution of the study groups according to demographic variables.

Demographic variables		Rheumatoid Arthritis group N (%)	Control group N (%)
Age (years)	Mean ± S.D	55.0 ± 10.22	38.57 ± 11.51
Gender	Males	12 (28.0)	19 (40.4)
	Females	31 (72.0)	28 (59.6)
Total	90 (100.0)	43 (47.8)	47 (52.2)

*p* = 0.21.

Mean disease duration of RA group was 2 years (range 1–5 years). All of the RA patients were under combination drugs [Steroids, NSAIDS and Disease Modifying Anti-rheumatic Drugs (DMARDS)]. These medications adversely affected unstimulated salivary flow (p < 0.001), stimulated salivary flow (p < 0.001) and pH (p < 0.001).

The RA group produced significantly less stimulated and unstimulated saliva than the control group did. In comparison to the control group, the salivary pH was significantly less in the RA group. (Table 2).

**Table 2:** Salivary flow and pH among study groups.

Parameter	Rheumatoid Arthritis group	Control group	*p* value
**Unstimulated Flow (mL/min)**	0.11 ± 0.05	0.23 ± 0.24	**<0.001**
**Stimulated Flow (mL/min)**	0.69 ± 0.19	1.16 ± 0.38	**<0.001**
**Salivary pH Normal (%)**	1 (2.3 %)	23 (49.0 %)	**<0.001**
**Salivary pH Low (%)**	42 (97.7 %)	24 (51.0 %)	

In the RA group, the mean number of decayed teeth (DT) and DMFT was significantly higher.

(DMFT: 14.14 ± 5.82, DT: 8.56 ± 5.17) than the control group (DMFT: 9.20 ± 6.63, DT: 4.53 ± 4.64) (p < 0.001). The mean number of filled (FT) and missing (MT) teeth in the RA group (MT: 2.74 ± 5.34, FT: 2.84 ± 4.54) and the control group (MT: 2.51 ± 4.66, FT: 2.16 ± 3.73) (p = 0.70 and p = 0.91 respectively) did not differ significantly. Mean CPI score was significantly higher in RA group (Gingival bleeding: 0.67 ± 0.14, Pocket: 0.61 ± 0.18) than the control group (Gingival bleeding: 0.27 ± 0.19, Pocket: 0.19 ± 0.14) (p < 0.001). (Table 3).

**Table 3:** Dental and Periodontal Health among study groups.

Parameter	Rheumatoid Arthritis group	Control group	*p* value
**Caries (DMFT)**	14.14 ± 5.82	9.20 ± 6.63	**<0.001**
**Decayed Teeth (DT)**	8.56 ± 5.17	4.53 ± 4.64	**<0.001**
**Missing Teeth (MT)**	2.74 ± 5.34	2.51 ± 4.66	0.70
**Filled Teeth (FT)**	2.84 ± 4.54	2.16 ± 3.73	0.91
**Gingival Bleeding**	0.67 ± 0.14	0.27 ± 0.19	**<0.001**
**Pocket Depth**	0.61 ± 0.18	0.19 ± 0.14	**<0.001**

RA was found to be a significant predictor of periodontitis (OR: 2.8, 95 % CI: 1.6–5.0, p < 0.001) and dental caries (OR: 2.4, 95 % CI: 1.6–5.8, p < 0.001). Age was a powerful predictor among the covariates, with each year of age increase linked to increased risk of both periodontitis (OR: 1.26, p = 0.002) and dental caries (OR: 1.04, p = 0.005). Dental caries (OR: 3.2, p = 0.001) and periodontitis (OR: 2.5, p = 0.003) were both markedly elevated by decreased unstimulated salivary flow. The likelihood of periodontitis (OR: 2.1, p = 0.04) and dental caries (OR: 2.4, p = 0.02) was also linked to low salivary pH. The duration of RA illness was another significant predictor; the likelihood of acquiring periodontitis (OR: 1.3, p = 0.02) and dental caries (OR: 1.2, p = 0.03) increased with each extra year of illness duration. In terms of drug use, using steroids was substantially linked to higher risks of periodontitis (OR: 2.6, p = 0.01) and dental caries (OR: 2.3, p = 0.04). However, DMARDs (OR: 1.8, p = 0.07 for caries, OR: 1.9, p = 0.06 for periodontitis) and biologics (OR: 1.5, p = 0.12 for caries, OR: 1.6, p = 0.10 for periodontitis) did not have statistically significant association (Table 4).

**Table 4:** Association between Rheumatoid arthritis and Oral health status.

Variable	Dental Caries OR (95 % CI)	*p* value	Periodontitis OR (95 % CI)	*p* value
**Rheumatoid Arthritis**	2.4 (1.6–5.8)	**<0.001**	2.8 (1.6–5.0)	**<0.001**
**Age (Ref: per year increase)**	1.04 (1.01–1.09)	**0.005**	1.26 (1.12–1.49)	**0.002**
**Unstimulated Salivary Flow (Ref: Low)**	3.2 (1.9–5.4)	**0.001**	2.5 (1.4–4.6)	**0.003**
**Salivary pH (Ref: Low)**	2.4 (1.1–4.4)	**0.02**	2.1 (1.1–4.2)	**0.04**
**RA Disease Duration (Years)**	1.2 (1.0–1.4)	**0.03**	1.3 (1.1–1.5)	**0.02**
**Steroid Use**	2.3 (1.2–4.6)	**0.04**	2.6 (1.3–5.1)	**0.01**
**DMARDs Use**	1.8 (0.9–3.2)	0.07	1.9 (1.0–3.7)	0.06
**Biologics Use**	1.5 (0.7–3.0)	0.12	1.6 (0.8–3.2)	0.10

**Fig. 1:**
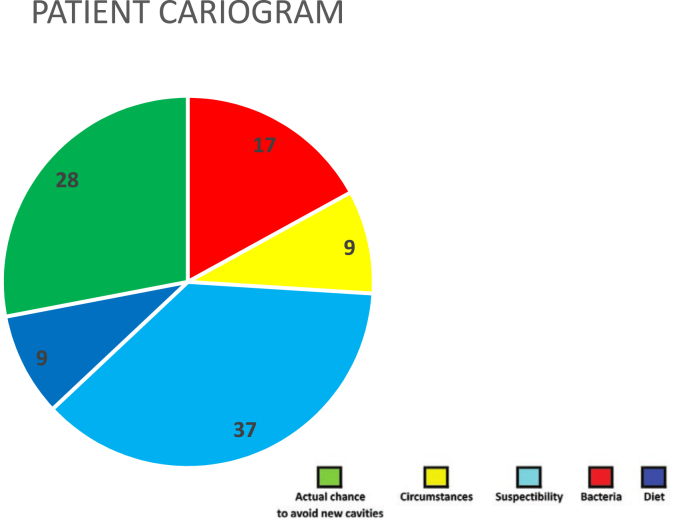
Rheumatoid Arthritis group.

**Fig. 2:**
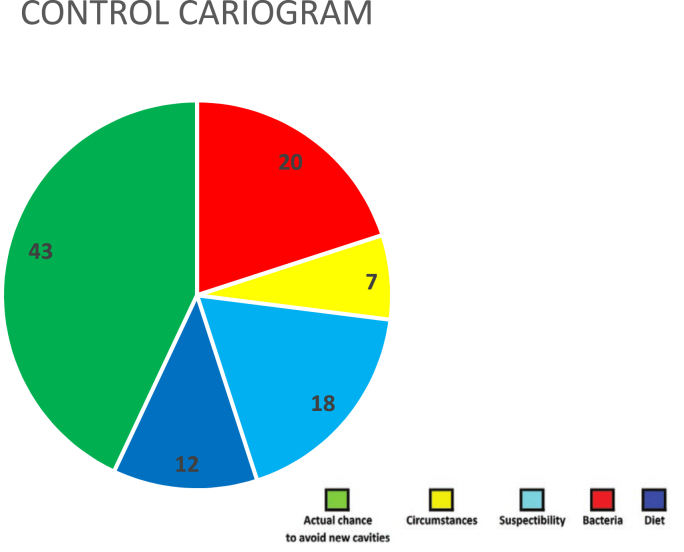
Control group.

Caries risk assessment done using Reduced Cariogram showed higher caries risk in RA group compared to controls. Actual chance to avoid new cavities (Green sector) was lower in RA group (28 %) when compared to controls (43 %). In RA patients, the bacterial component (Red sector) was 17 %, while it was 20 % in controls. Dietary component (Dark blue sector) was 9 % in RA patients, whereas in controls, it was 12 %. Susceptibility sector (Light blue sector) determined by saliva secretion and the fluoride programme was higher in RA group (37 %) when compared to controls (18 %). Circumstances (Yellow sector) which integrates systemic diseases and dental caries experience are much higher at 37 % in RA group whereas in controls, it accounted for 7 %. (Fig. 1, Fig. 2).

## 4 Discussion

Anti-citrullinated protein antibodies (ACPA) are a major factor in the pathophysiology of rheumatoid arthritis (RA), an autoimmune disease marked by persistent inflammation of the synovial joints ^18^. In addition to joint-related symptoms, RA can also show up in the oral cavity. In this context, we observed variations in salivary secretion, dental caries and periodontitis between RA patients and the control group. In fact, the data show that individuals with RA have significantly higher prevalence of periodontal disease (PD) than those without RA, which is consistent with the majority of the published literature.^13^, ^19^, ^20^, ^21^ Both PD and RA are characterized by inflammation and nearby bone loss. They also have comparable genetic and environmental risk profiles, with high correlations between smoking and genes involved in immune responses ^22^.

The RA group had measurably less stimulated and unstimulated saliva, which is consistent with two studies^5^, ^8^ that found a consistent relationship between salivary flow and RA. Similar to a study, a larger percentage of individuals in the RA group had lower salivary pH ^8^. As a result, patients with RA have significantly altered salivary parameters. The prevalence of PD was considerably higher in RA patients than in controls, which is in line with earlier research.^3^, ^4^, ^6^, ^18^, ^20^, ^21^ Given that both PD and RA share inflammatory pathways, bone loss mechanisms, and risk factors like smoking and genetic susceptibility, the relationship between the two diseases is well established ^21^. The necessity for focused periodontal treatments is further supported by our data, which show a significant burden of periodontal disease in RA patients with greater gingival bleeding scores and deeper periodontal pockets.

Due to the decreased salivary flow that RA disease causes, there is an increased risk of dental caries ^17^. In line with a few studies, the RA group experienced significantly more dental caries.^8^, ^16^, ^23^ Cariogram is a widely used model for caries prediction ^24^. Caries risk assessment was done using Cariogram which showed higher caries risk in RA when compared to controls. There were no studies done in RA patients available for comparison.

Multivariate logistic regression analysis revealed association between RA and oral health. Even after controlling for age, salivary characteristics, and medication use, RA patients were still at a considerably greater risk of developing periodontitis and dental caries. Age stood out among the confounders as a powerful predictor, highlighting the cumulative effect of aging on dental health. Furthermore, reduced unstimulated salivary flow was a significant risk factor for periodontitis and dental caries, highlighting the importance of saliva in preserving oral health. Additionally, a lower salivary pH was linked to a higher incidence of both disorders. It is noteworthy that the duration of RA disease was directly correlated with the decline of oral health, indicating that the advancement of RA raises the risk of periodontitis and dental cavities.

The present study has certain strengths and limitations. To the best of our knowledge, the current study is the first of its kind to do caries risk assessment in RA using cariogram. Salivary parameters were assessed using standard kits reflecting high internal validity. The study has its limitations. Although a cross-sectional study design suggests the existence of risk factors, it does not permit the assessment of causality between study variables. It is possible that unidentified systemic disease was present and that this affected the salivary parameters. Risk assessment may have been impacted by the omission of variables such as the *Streptococcus mutans* count. A more thorough understanding of caries risk in RA patients may be possible with future research that takes these aspects into account. In addition, the study setting and regional differences should be taken into account because access to dental healthcare facilities and dietary habits may affect the assessment of caries risk and the generalizability of the results. Future studies should concentrate on longitudinal research to evaluate how salivary parameters change over time and how long-term use of RA drugs affects oral health. Dietary advice and customized dental care regimens may be incorporated into RA treatment to assist reduce oral health issues in this susceptible group.

## 5 Conclusion

This study highlights the adverse impact of RA on oral health, emphasizing increased dental caries prevalence and compromised salivary profile in RA patients These findings underscore the importance of comprehensive oral health assessments and tailored preventive strategies for RA patients to mitigate oral health complications. Prospective research is advised to comprehend changes in salivary parameters and the long-term effects of medications in RA patients.

## Data availability

The corresponding author can provide the datasets used and/or analyzed for this study upon reasonable request.

## Funding

None.

## Declaration of competing interest

The authors declare that they have no known competing financial interests or personal relationships that could have appeared to influence the work reported in this paper.
